# Virus replicon particle vaccines expressing nucleoprotein of influenza A virus mediate enhanced inflammatory responses in pigs

**DOI:** 10.1038/s41598-017-16419-w

**Published:** 2017-11-27

**Authors:** Meret E. Ricklin, Sylvie Python, Nathalie J. Vielle, Daniel Brechbühl, Beatrice Zumkehr, Horst Posthaus, Gert Zimmer, Nicolas Ruggli, Artur Summerfield

**Affiliations:** 1 Institute of Virology and Immunology IVI, Sensemattstrasse 293, Mittelhäusern, Switzerland; 2 Institute for Animal Pathology, Vetsuisse Faculty, University of Bern, Länggasstrasse 122, Bern, Switzerland; 3 Department of Infectious Diseases and Pathobiology, Vetsuisse Faculty, University of Bern, Länggasstrasse 122, Bern, Switzerland

## Abstract

Studies in the mouse model indicate that the nucleoprotein of influenza A virus represents an interesting vaccine antigen being well conserved across subtypes of influenza virus but still able to induce protective immune responses. Here we show that immunizations of pigs with vesicular stomatitis virus- and classical swine fever virus-derived replicon (VRP) particles expressing the nucleoprotein (NP) of H1N1 A/swine/Belzig/2/01 induced potent antibody and T-cell responses against influenza A virus. In contrast to a conventional whole inactivated virus vaccine, the VRP vaccines induced both NP-specific CD4 and CD8 T cells responses, including interferon-γ and tumor-necrosis-factor dual-secreting cell. Although T-cells and antibody responses were cross-reactive with the heterologous H1N2 A/swine/Bakum/R757/2010 challenge virus, they did not provide protection against infection. Surprisingly, vaccinated pigs showed enhanced virus shedding, lung inflammation and increased levels of systemic and lung interferon-α as well as elevated lung interleukin-6. In conclusion, our study shows that NP, although efficacious in the mouse model, appears not to be a promising stand-alone vaccine antigen for pigs.

## Introduction

Vaccination plays an important role in protecting humans and animals against influenza A virus (IAV) infection. However, a main problem of IAV vaccines is the correct selection of the vaccine strain. IAV have an extraordinary ability to escape immunological memory by adaptive mutations (antigenic drift) and by exchanging genomic segments (antigenic shift). This results in a lack of population immunity and regular emergence of new IAV epidemics making adaptation of vaccine antigenic components on a regular basis necessary. The hemagglutinin (HA) and the neuraminidase (NA) represent the most protective viral antigens, but as a consequence also have the highest antigenic variability. For these reasons, many approaches have been taken to identify antigenically conserved regions in the different internal proteins of IAV such as NP, M1 and M2. NP represents a structural multifunctional protein, which forms complexes with the viral RNA protecting it from degradation, but in contrast to HA and NA, is not found in the viral envelope.

A multitude of studies in the mouse model have demonstrated the potential of NP as a vaccine candidate (for review see^1^). As early as in 1987, a study employed purified NP to induce partial protection in mice^2^. Since then, many vaccination approaches confirmed NP as an interesting vaccine candidate, at least in the murine model. For DNA vaccines, protection was shown to be cell-mediated^3^ but was not always found to be efficacious^4^. Therefore, prime-boost vaccination approaches combining priming with a DNA vaccine followed by a boost with a viral vector have been tested and demonstrated to induce superior levels of immunity^5,6^. As expected, also viral vectors expressing NP including adenovirus, modified virus Ankara (MVA), parainfluenza virus and Pichinde virus have been used and shown to induce protection or partial protection in mice^7–10^.

In line with the fact that NP contains many CD8 T-cell epitopes^11^, several studies using the murine model pointed to an important role of T cells in the protection induced by NP-based vaccines^3,5,7^. Nevertheless, also antibodies against NP were shown to play a role in protecting mice from IAV challenge^12–14^. As NP is not expressed on the surface of IAV virions but on the surface of infected cells^15,16^, antibody-dependent cellular cytotoxicity has been proposed as an immunological mechanism of protection^14,17^.

The efficacy of NP-based IAV vaccines was also tested in other species, however, with conflicting results. In chickens, vaccination with a fowlpox vector expressing NP did not mediate any measurable protection^18^. Priming with a recombinant adenovirus *in ovo* and boosting with recombinant MVA vector was only able to reduce cloacal shedding of a low-pathogenic avian IAV^19^. In ponies, DNA vaccine priming followed by MVA booster vaccination conferred partial protection^6^. In ferrets, a DNA vaccine only induced “some benefits” compared to unvaccinated animals^20^. While a vaccination regime composed of three injections of a DNA vaccine followed by a boost with an adenovirus expressing NP and M2 was protective in one study^21^, another study reported no positive effect^22^. The protective value for using NP-based vaccines in pigs is also unclear. In early work, enhanced disease was found after vaccination of pigs with a DNA vaccine expressing M2, which was even aggravated if the vaccine expressed both M2 and NP^23^. In contrast, an adenovirus vector expressing NP reduced nasal shedding and lung disease scores on day five post infection^24^, and an alphavirus vector decreased viral shedding and reduced viral lung load in pigs^25^. In humans, a preliminarily assessment of the protective value of an MVA vector expressing NP and M1 did not permit a clear conclusion. In the control group 5 out of 10 patients got influenza-like symptoms following challenge infection whereas in the vaccinated group it was 2 out of 10 patients^26^.

All these studies indicate that the protective value of NP as a vaccine antigen depends on the species, the vaccine delivery system and possibly also the challenge virus. Considering this, the present study was initiated with the aim of clarifying the value of an NP-based vaccine in pigs using two different virus replicon particle (VRP) systems. A cytotoxic VRP based on vesicular stomatitis virus (VSV)^27,28^ and a non-cytotoxic VRP based on classical swine fever virus (CSFV)^29,30^ were compared with whole inactivated influenza virus (WIV). The VRP were selected based on their potent ability to induce both B and T cell responses in pigs. Surprisingly, although both types of VRP induced a potent anti-NP antibody and T-cell responses, no protection was observed during the acute phase of the disease.

## Results

### VRP vectors mediate expression of NP in infected cells

**Figure 1:**
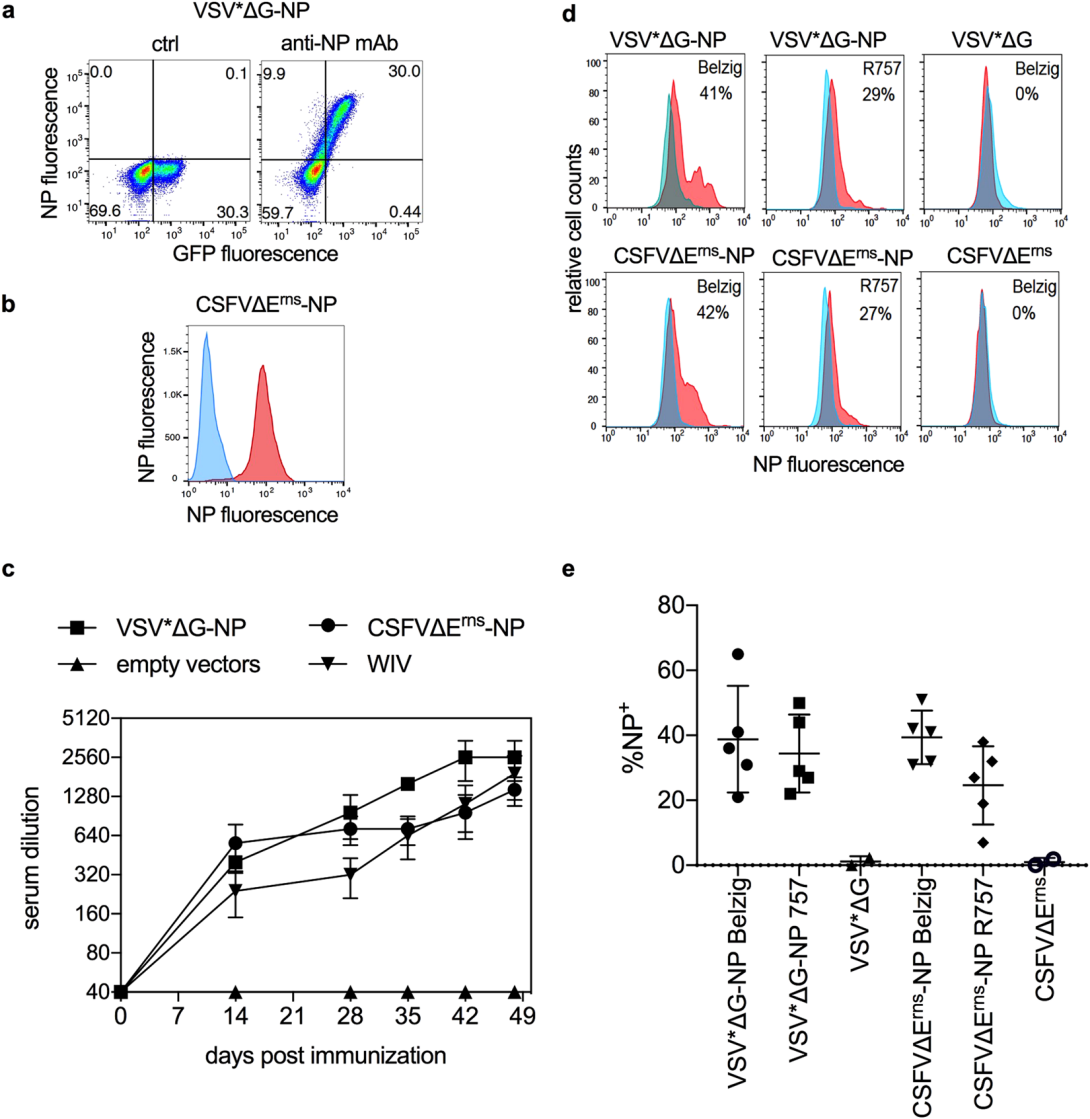
VRP vector mediated NP expression and induce NP-specific antibodies in pigs. (**a**) BHK-21 cells infected by VSV*ΔG-NP express NP. The left plot demonstrates GFP expression only after infection with VSV*ΔG, and the right plot GFP/NP double positive cells after infection with VSV*ΔG-NP. (**b**) Overlay histogram of SK-6 cells infected with CSFVΔE^rns^ (blue histogram) and CSFVΔE_rns_-NP (red histogram). (**c**) Kinetics of NP antibody responses induced by VRP and WIV vaccines determined by ELISA. Pigs were vaccinated with either VSV*ΔG-NP, CSFVΔE_rns_-NP, empty vectors or WIV vaccines, and immune sera collected at different time points and tested by ELISA. Titers were determined as the highest serum dilution leading to photometric values above the detection threshold. The pigs received a booster immunization at day 28. (**d**) Reactivity of sera collected at day 48 after VRP vaccination with MDBK cells infected with Belzig (H1N1, homologous virus), R757/10 (H1N2, heterologous challenge virus) or mock. In the upper row, representative results for sera from the VSV VRP vector-immunized pigs, and in the lower row results from the CSFV VRP-immunized animals are shown. The blue histograms represent mock-infected cells, and is overlaid with the red histograms representing the reactivity with virus-infected cells. (**e**) Reactivity of all sera from VSV*ΔG-NP, CSFVΔE^rns^-NP and empty vectors injected pigs with Belzig (H1N1)- or R757/10 (H1N2)-infected MDCK cells, determined as described in Fig. 1d. Statistical significant differences between two groups were calculated using unpaired one-way ANOVA followed by Tukey’s multiple comparisons test (***P < 0.0001; **P < 0.005; *P < 0.05).

Figure 1a demonstrates functional expression of NP derived from Belzig/01 (H1N1) influenza virus. after infection of BHK-21 cells with VSV*ΔG-NP VRP. Both the empty vector and the VSV*ΔG-NP also expresses GFP, but only VSV*ΔG-NP-infected cells express NP, which was exclusively found in the GFP^+^ population. Also, CSFVΔE^rns^-NP infected SK-6 cells expressed high level of NP as shown in Fig. 1b. Considering these results, the VRP vaccine candidates were tested *in vivo*.

### VRP and WIV vaccines induce high level of anti-NP antibodies

After the first vaccine injection, all pigs seroconverted by day 14 post vaccination (p.v.; Fig. 1c). At days 28, 35 and 42 p.v., pigs vaccinated with VSV*ΔG-NP had significantly higher levels of NP-reactive antibodies than WIV vaccinated animals (p = 0.003, p < 0.0001and p = 0.0068, respectively). Nevertheless, on day 48 no significant differences were found between the groups. The VRP-vaccine-induced antibodies cross-reacted with the selected challenge virus R757/10 (H2N1), when analyzed on homologous Belzig (H1N1)- and heterologous R757 (H2N1)-infected MDCK cells by flow cytometry (Fig. 1d). When all sera collected at day 48 were analyzed no significant difference in their reactivity with Belzig (H1N1)- and R757 (H2N1)-infected cells was found in terms of the percentage of infected cells (Fig. 1e). We also did not find significant differences in the reactivity of sera from VSV*ΔG-NP and from CSFVΔE^rns^-NP vaccinated pigs, confirming the ELISA results.

### NP-based VRP vaccines but not WIV induce virus-specific IFN-γ/TNF double producing CD8 T cells

**Figure 2:**
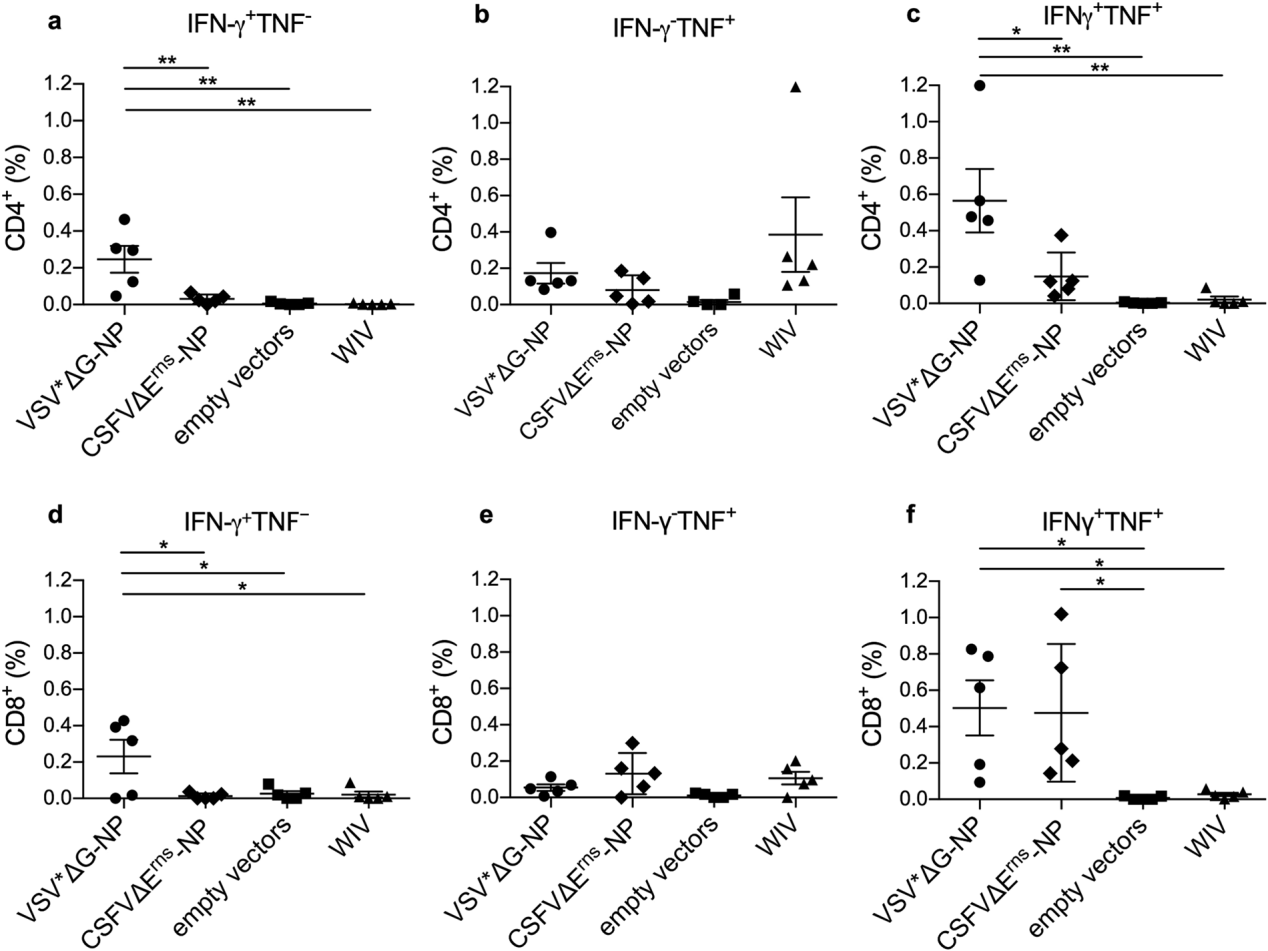
Peripheral blood CD4 and CD8 T-cell responses induced by VRP and WIV vaccines. PBMC were isolated at day 48 post vaccination (20 days after the booster injection) and re-stimulated *in vitro* with CAF or R757/10 (H1N2) virus to determine the percentage of virus-specific IFNγ (**a**,**d**), TNF (**b**,**e**) and dual cytokine (**c**,**f**) producing T cells in both the CD4 (A–C) and the CD8 (**d**–**f**) T cell subset. Asterisks indicate significant differences between two groups as calculated using unpaired one-way ANOVA followed by Tukey’s multiple comparisons test (***P < 0.0001; **P < 0.005; *P < 0.05).

In order to assess the level of vaccine-induced cell-mediated immunity present at the day of challenge, PBMC obtained on day 48 p.v. were restimulated *in vitro* for 16 h with R757/10 (challenge virus) or mock (CAV) to determine virus-specific IFN-γ, TNF and IFN-γ/TNF dual cytokine producing CD4 and CD8 T cells (Fig. 2). A representative flow cytometry analysis is shown in Supplementary Fig. 1, which also shows that the T-cell reacted to both homologous and heterologous virus. Our data indicate that the recombinant VSV was the most potent vector in inducing CD4 T cells. Only with this vaccine, a significant increase of virus-specific IFN-γ and IFN-γ/TNF double-cytokine producing CD4 T cells compared to the empty vector control was found (p = 0.0016 and 0.0029, respectively; Fig. 2a–c). With respect to CD8 T cells, VSV*ΔG-NP was the only vaccine able to induce detectable numbers of IFN-γ producing cells (p = 0.0353; Fig. 2d). However, both VRP-based vaccines induced IFN-γ/TNF producing CD8 T cells, when compared to the empty vector controls (VSV: p = 0.0332 and CSFV: p = 0.046; Fig. 2f). The frequency of these dual-functional T cells was above that of single cytokine producing cells, further supporting the potent ability of both VRP-based vaccines to induce virus-specific CD8 T cells.

### NP-based VRP vaccines do not prevent the induction of fever by IAV

**Figure 3:**
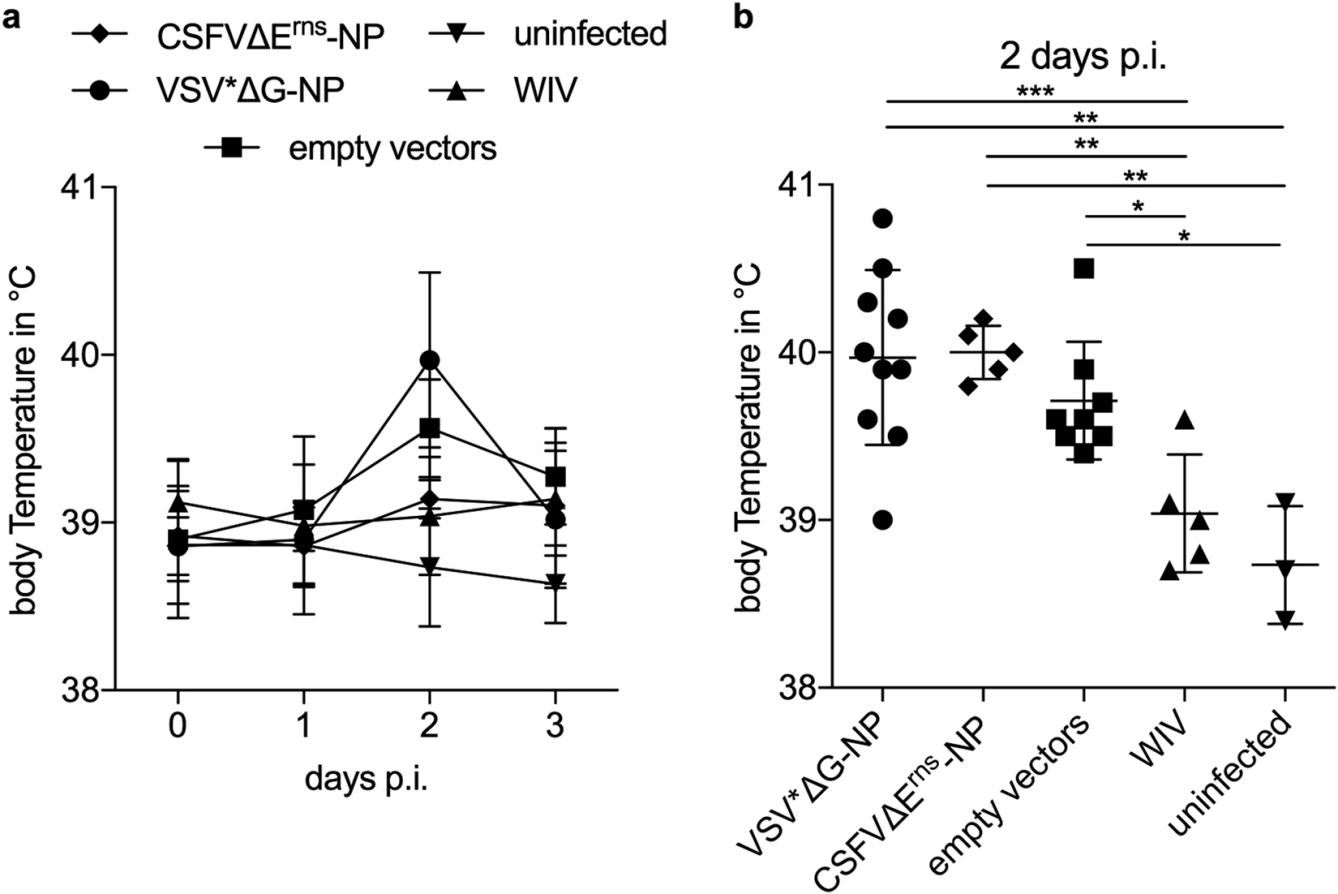
Body temperature following heterologous challenge. VSV*ΔG-NP, CSFVΔE_rns_-NP, empty vectors or WIV-vaccinated animals were challenged with R757/10 (H1N2). In (**a**), mean values and standard deviations of body temperatures are shown for each group. In (**b**), values for individual animals are shown for day 2 p.i. Asterisks indicate significant differences between two groups as calculated using unpaired one-way ANOVA followed by Tukey’s multiple comparisons test (***P < 0.0001; **P < 0.005; *P < 0.05).

The protective value of the NP-based vaccines was tested with a heterologous challenge model in which a conventional WIV induced no or very little protection^28^. We hypothesized that in this model cross-reactive anti-NP humoral and cellular immunity should provide a measurable level of protective immunity. In the absence of secondary bacterial infections, swine influenza is usually a mild disease which is rapidly controlled by the host. In line with this and our previous experimental work^28^, no coughing nor increased breathing were observed in SPF pigs during the first three days after challenge infection. Pigs vaccinated with control VRP vectors or with VRPs expressing NP showed a mild and transient increase in body temperature at 2 days post infection (p.i.), whereas uninfected pigs or pigs vaccinated with the WIV did not show significantly elevated body temperatures (Fig. 3). There was no statistically significant difference between VRP expressing the NP and the empty vector controls.

### NP-based VRP vaccines cause increased IAV-mediated lung lesions

**Figure 4:**
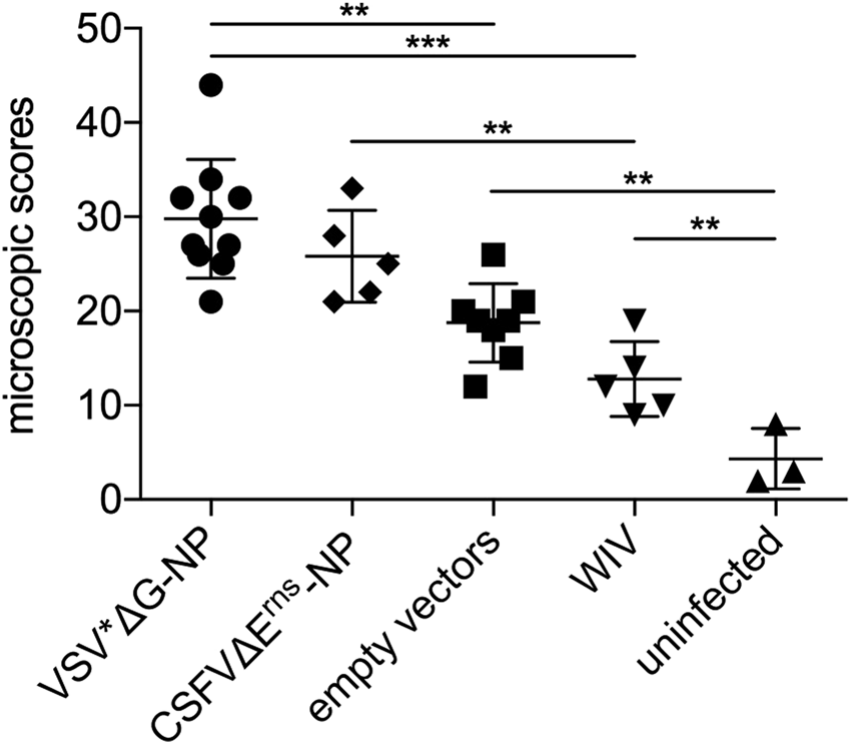
Lung lesions after challenge infection. Lung lesions were assessed microscopically from each lobe at three days p.i. with R757/10 (H1N2). Scores were determined in a blinded manner by an even-handed pathologist for each lobe, and pooled to one value per animal. Asterisks indicate significant differences between two groups as calculated using unpaired one-way ANOVA followed by Tukey’s multiple comparisons test (***P < 0.0001; **P < 0.005; *P < 0.05).

When the lungs of pigs were analyzed microscopically three days p.i., all infected pigs had significantly more lesions that uninfected animals (Fig. 4). Surprisingly, we found increased lesion scores in the VSV*ΔG-NP-vaccinated animals when compared to animals vaccinated with either the control vectors or WIV (p = 0.0008 and p < 0.0001, respectively). Furthermore, the CSFVΔE^rns^-NP vaccinated pigs had significantly more severe lesions when compared to the WIV group (p = 0.0031).

### NP-based VRP vaccines cause increased viral RNA levels in oronasal swabs

**Figure 5:**
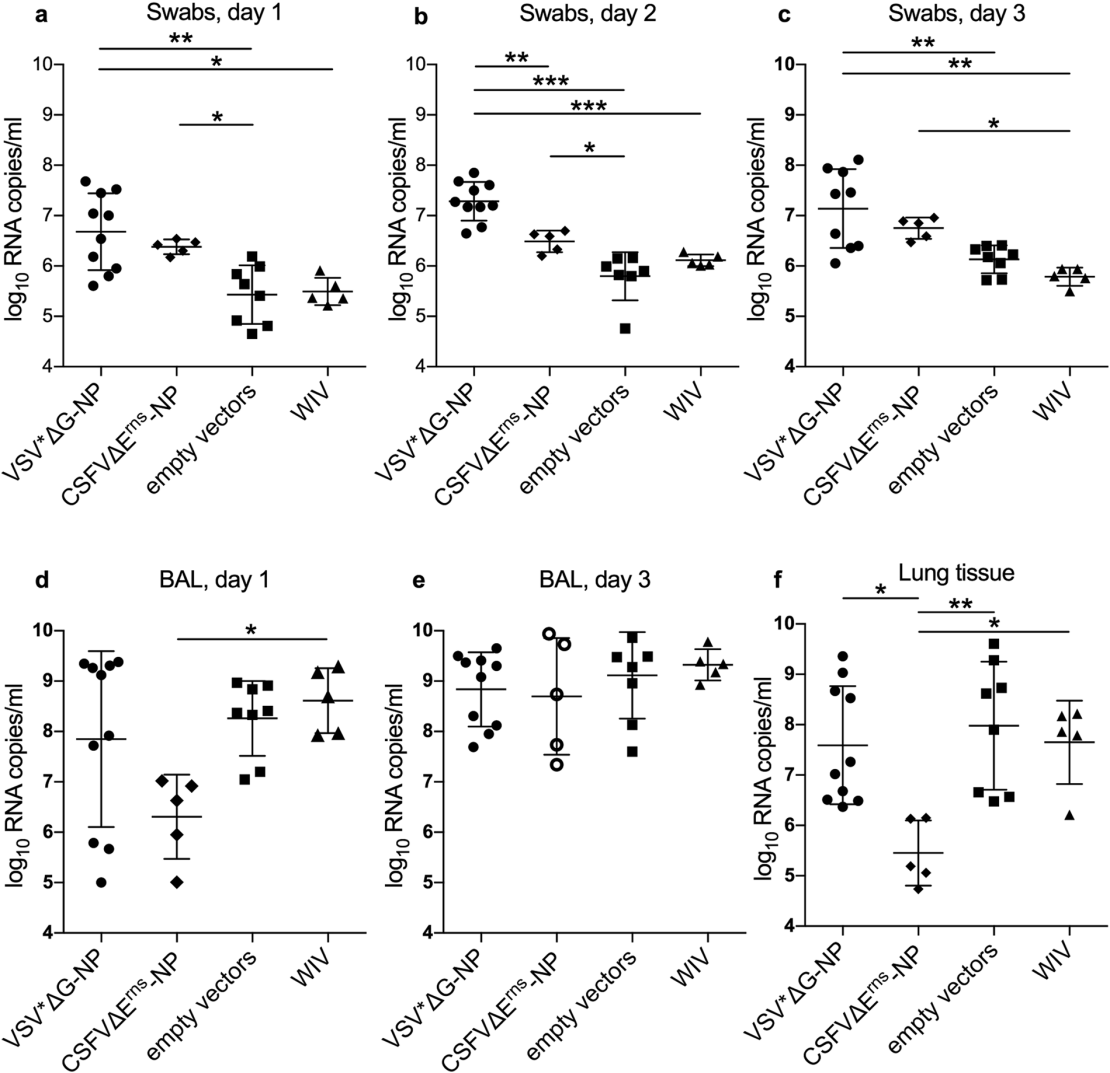
Viral RNA loads after challenge infection. Viral RNA copies/ml were quantified by RT-qPCR in swabs on days one (**a**), two (**b**) and three (**c**), in BAL fluid on day one (**d**) and day three (**e**), and in lung tissue on day three (**f**) p.i. with R757/10 (H1N2). Asterisks indicate significant differences between two groups as calculated using unpaired one-way ANOVA followed by Tukey’s multiple comparisons test (***P < 0.0001; **P < 0.005; *P < 0.05).

In oronasal swabs collected on days 1, 2 and 3 p.i., more viral RNA was found in animals that had been immunized with the VSV*ΔG-NP vaccine. This observation was made when the VRP groups were compared with either the control vector group or the WIV group (Fig. 5 a to c; p values for the comparison to empty vectors were p = 0.0006, p < 0.0001 and p = 0.0018 on days 1, 2 and 3, respectively). Also, the CSFVΔE^rns^-NP vaccinated animals secreted more virus in their oronasal fluids on day 1 and 2 p.i, when compared to the empty vector controls (p = 0.042 and 0.0158, respectively). However, these differences were not found when BAL fluids were analyzed (Fig. 5d and e). In lung tissue, it appeared that the CSFVΔE^rns^-NP vaccinated pigs had reduced levels of RNA (p = 0.0022; Fig. 5f). However, the levels of viral RNA varied considerably within the groups. This variability could have been caused by a not uniformly distributed virus replication in the pig lung and the fact that we always sampled from the same area of the cranial lobe as macroscopical lesions were difficult to see.

### NP-based VRP vaccines cause increased IFN-α levels following IAV challenge

**Figure 6:**
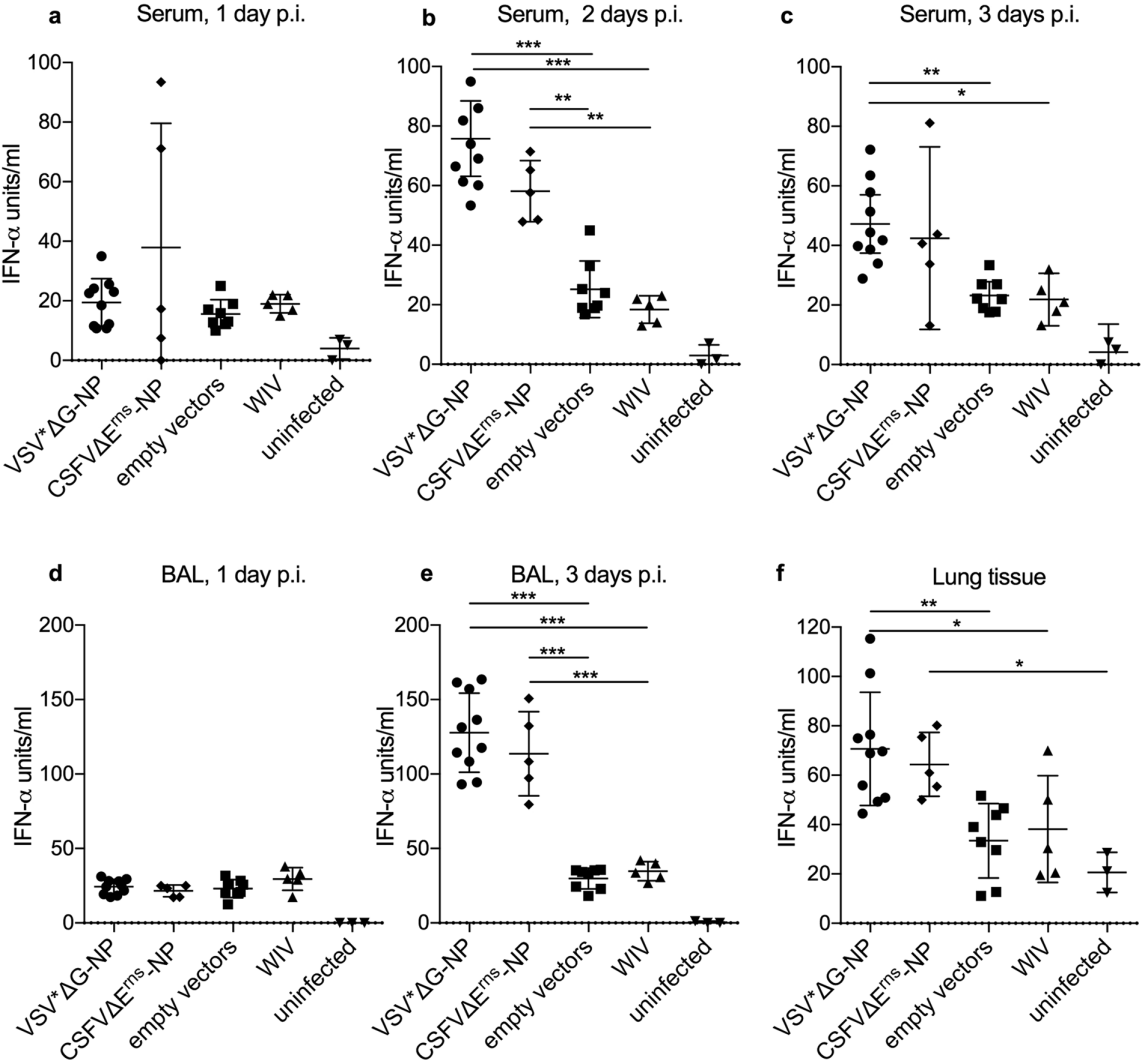
*In vivo* IFN-α responses after challenge infection. IFN-α levels were determined by ELISA in serum of infected animals on days one (**a**), two (**b**) and three (**a**), in BAL fluid on day one (**d**) and day three (**e**), and in lung tissue on day three (**f**) p.i. with R757/10 (H1N2). Asterisks indicate significant differences between two groups as calculated using unpaired one-way ANOVA followed by Tukey’s multiple comparisons test (***P < 0.0001; **P < 0.005; *P < 0.05).

In order to characterize the innate and inflammatory immune response, we investigated IFN-α levels in serum, BAL fluid and lung tissue by ELISA. The challenge infection induced a systemic IFN-α in all pigs reaching maximum levels on day 2 p.i. (Fig. 6b,c). At this time point, animals vaccinated with VSV*ΔG-NP or CSFVΔE^rns^-NP had clearly higher levels of systemic IFN-α when compared to the empty vector (p < 0.0001 and p = 0.0007, respectively) or the WIV group (p < 0.0001 and p = 0.0002, respectively). On day 3 p.i., only the IFN-α levels of the VSV*ΔG-NP group were significantly different from the empty vector controls (p = 0.0062). In the BAL fluid collected at day 1 p.i., we found elevated IFN-α in all samples from infected pigs (p < 0.0001) but no significant difference between the vaccine groups. On day 2 p.i., as with the systemic response, increase IFN-α levels were found with all infected groups. The animals which received either the VSV*ΔG-NP or CSFVΔE^rns^-NP vaccine had clearly elevated IFN-α (p < 0.0001; Fig. 6e). In lung tissues, only the VSV*ΔG-NP had significantly higher IFN-α when compared to the empty vector controls (p = 0.0022; Fig. 6f). The CSFVΔE^rns^-NP group had elevated IFN-α levels but this did not reach the threshold of statistical significance when compared to the empty vector controls (p = 0.0514).

### NP-based VRP vaccines cause increased IL-6 levels in the lung after IAV challenge

**Figure 7:**
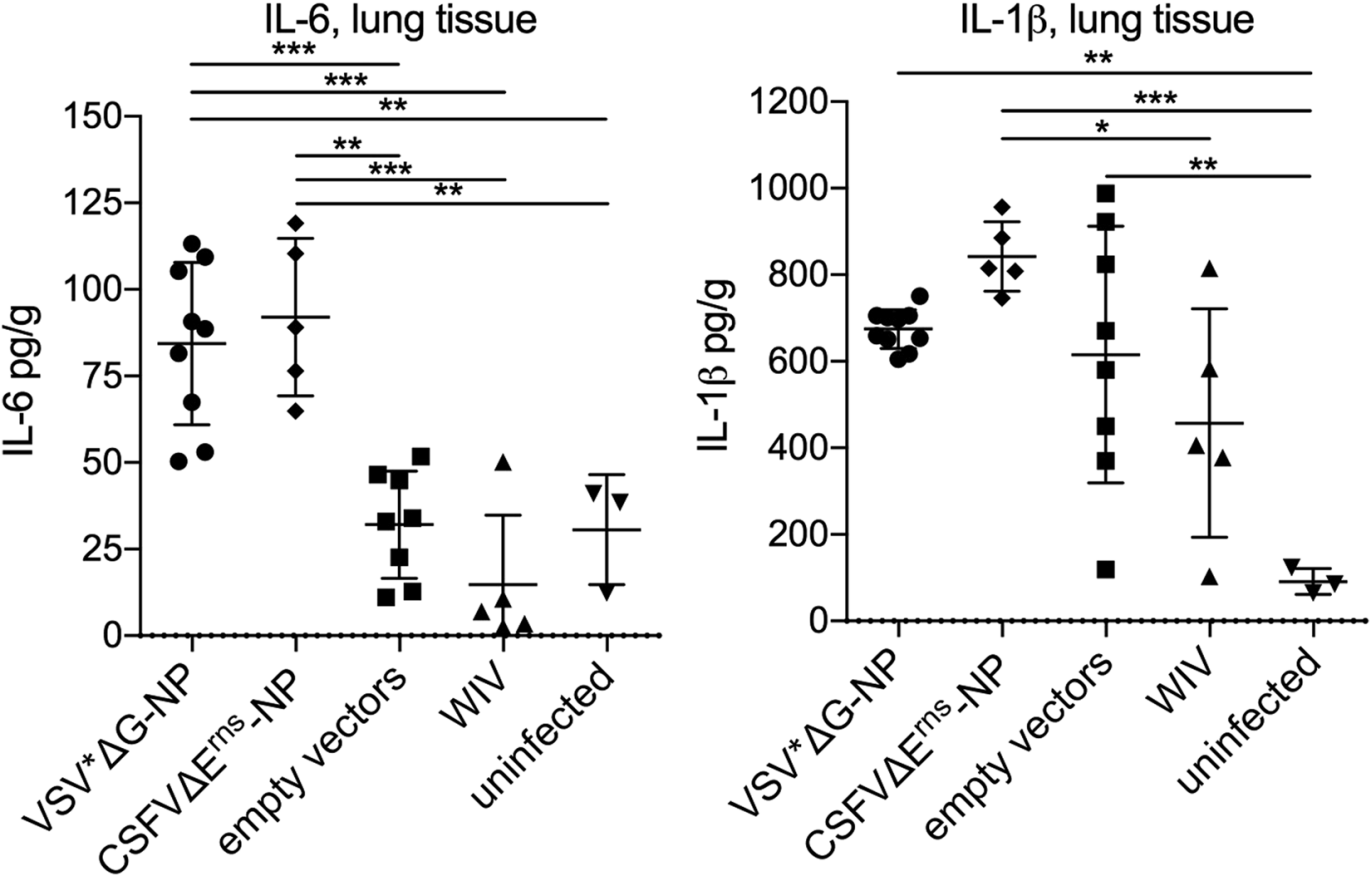
*In vivo* IL-6 and IL-1β responses in the lung after heterologous challenge. Levels of the inflammatory cytokines IL-6 (**a**) and Il-1β (**b**) were determined by ELISA in lung homogenates prepared on day three p.i. with R757/10 (H1N2). Asterisks indicate significant differences between two groups as calculated using unpaired one-way ANOVA followed by Tukey’s multiple comparisons test (***P < 0.0001; **P < 0.005; *P < 0.05).

In view of the elevated IFN-α levels, we also tested the collected samples for the proinflammatory cytokines IL-6 and IL-1β. In none of the sera and BAL fluids elevated levels of these cytokines were found, reflecting the mild disease observed (data not shown). Nevertheless, animals vaccinated with either VSV*ΔG-NP or CSFVΔE^rns^-NP had enhanced levels of IL-6 in lung tissue, which was not observed with animals that had been vaccinated with the empty VRPs or WIV (p = 0.0001 and p = 0.0002, respectively; Fig. 7a). All three groups receiving the VRP vaccines had elevated levels of IL-1β in lung tissue when compared to unvaccinated pigs (Fig. 7b). IL-1β was found to be highest in the CSFVΔE^rns^-NP group, although no statistically significant difference compared to the empty vector controls was found (Fig. 7b).

## Discussion

The present study demonstrates in the pig model that immunization with VRPs encoding the NP antigen has no protective value in a heterologous IAV challenge setting, despite the induction of high levels of anti-NP antibodies and dual-functional IFN-γ/TNF secreting CD4 and CD8 T cells. We rather found signs of enhanced inflammation in terms of increased microscopic lung lesions, increased virus shedding through oronasal secretions, and increased levels of IFN-α and IL-6.

Our work is in line with the work of others demonstrating that vaccination against internal proteins can cause enhanced inflammation without obvious protection^23,31^, although these studies did not employ NP as the sole vaccine antigen. The validity of our findings is underlined by the fact that two different VRP vaccines gave comparable results. The VSV-based VRP is highly cytopathogenic and results in very high but short-lived antigen expression levels. Although the glycoprotein (G) gene has been deleted from the viral genome, the protein is present on the surface of the trans-complemented VRPs mediating infection of cells in the muscle following intramuscular application (immunization via the intradermal route is less efficient)^32^. In contrast, CSF-VRP are non-cytopathogenic and therefore provide long-lasting protein expression^30^. CSF-VRP are more efficacious if applied via the intradermal route compared to intramuscular injection^29^. The surface proteins of CSFV mediate macrophage and DC targeting^33,34^. This is interesting in view of recent results indicating that a vaccine composed of recombinant NP, M2e and HA2 proteins can also lead to disease exacerbation in pigs when targeted to DC following intradermal application^31^. Generally, our data indicated that vaccine-induced enhanced inflammation was more prominent with the VSV-VRP. With this vaccine, we found more readouts to be statistically significant when compared to the empty vector controls. This included viral RNA in the swabs, microscopic lung scores, systemic and lung IFN-α responses, but not local IL-6 responses. It is possible that these differences are attributed to more potent CD4-T-cell responses induced by the VSV vaccine. Only this vaccine induced statistically significant CD4 T-cell responses measurable in the peripheral blood.

Our results contrast with works done using adenovirus and alphavirus vector-based NP vaccines, which suggest that NP-based vaccines can also provide beneficial effects in pigs^24,25^. The reasons for this discrepancy are unclear but are possibly related to a different challenge model or differences in the experimental system. In order to clarify this, a side-by-side testing of these vaccines would be necessary.

Independently of this ambiguity, it is clear that vaccines based on NP alone are not efficacious in pigs as opposed to mouse models. In ferrets, which are also considered to be a good model for human influenza, NP-based vaccines have been disappointing when compared to the results obtained in the mouse model^20,22^. It is therefore likely that human vaccines that are based solely on internal influenza virus proteins might not hold the expectations provoked by the data obtained with mice.

Nevertheless, it is important to note that beneficial effects of anti-NP immunity based on the induction of cytotoxic CD8 effector cells would be expected to be more important at later stages of the disease as these cells essentially participate in the clearance of infected cells. Unfortunately, we cannot comment on such a positive effect of NP-based immunity as our study was terminated in the acute phase of the disease. Although CD8 effector T cells are playing a protective role in human influenza disease^35^, our data would indicate that they are non-protective in the acute phase of the disease or could even enhance inflammatory responses, at least in pigs.

Alternatively, or in addition, NP antibodies could also mediate enhanced inflammation and cytokine responses. NP is detected on the surface of infected cells^15,16^, and can mediate antibody-dependent cellular cytotoxicity and NK cell activation^14,17^. Nevertheless, it is possible that similar to the T cell response, NP-specific antibodies could also mediate protective effects when accompanied with responses against other viral proteins, in particular HA. This would explain why the WIV also inducing NP antibodies did not prime the immune system for enhanced inflammatory responses following challenge.

Taken together, the present study indicates that an NP stand-alone vaccine is not protective in a large animal model representing a natural IAV host, and a valuable model for human influenza^36^. Our studies as well as the previously published studies on potential “universal” influenza vaccines show that extrapolations from one species to another can by risky.

## Methods

### Cells

BHK-21 cells (ATCC, Manassas, VA, US) were grown in Glasgow Earle’s minimal essential medium (GMEM) (Life Technologies) supplemented with fetal bovine serum (FBS) (Biowest, Nuaillé, France). BHK-G43, a transgenic BHK-21 cell clone expressing the VSV G protein, was maintained as described previously^37^. Madin-Darby canine kidney (MDCK; ATCC) cells were propagated in MEM supplemented with 10% FBS, nonessential amino acids (Life Technologies), and 1mM sodium pyruvate (Life Technologies). The porcine kidney cell line SK-6 and the replicon packaging cell line SK-6(E^rns^)^29^ were cultured in MEM supplemented with 7% horse serum (SVA, Håtunaholm, Sweden). The SK-6(E^rns^) medium was supplemented with 0.25 mg/ml G418 (Calbiochem).

### Viruses

The influenza A viruses (IAV) used were A/swine/Belzig/2/01 (H1N1) (Belzig/01) and A/swine/Bakum/R757/2010 (H1N2) (R757/10), both kindly provided by the Friedrich-Loeffler-Institute, Greifswald-Insel Riems, Germany. IAV were propagated in 10-day-old embryonated specific-pathogen-free (SPF) chicken eggs. Following incubation of the infected embryos for 2 days at 37 °C, the allantoic fluid was collected and IAV titrated on confluent MDCK cells in the presence of 1 μg/ml of acetylated trypsin (Sigma-Aldrich). The cells received new medium at 24 h post infection (p.i.) and at 48h p.i. were fixed with 4% paraformaldehyde (Polysciences, Warrington, PA, USA) followed by permeabilization with PBS 0.3% saponin (Sigma-Aldrich) in presence of NP antibody (HB-65, ATCC) for 15 min. Then, horseradish peroxidase-conjugated goat anti-mouse antibody (Dako, Baar, Switzerland) was added, and a final color reaction using 3-amino-9-ethylcarbazole (AEC, Sigma-Aldrich) and H_2_O_2_ as substrate. Titers were calculated using the Reed and Muench formula^38^.

### Plasmids

The cDNA encoding NP of A/swine/Belzig/2/01 (H1N1) was kindly provided by Drs. Jürgen and Olga Stech (Friedrich-Loeffler-Institute, Riems, Germany)^39^. The NP gene was amplified by PCR and flanked by appropriate restriction endonuclease sites for cloning into the VSV and CSFV replicon plasmids. For the VSV vector, the NP gene was inserted into the MluI and BstEII endonuclease restriction sites of the pVSV* plasmid placed upstream and downstream of the fourth transcription unit of the VSV genome^27^. A cDNA encoding enhanced green fluorescent protein (eGFP) was inserted into an additional transcription unit located in the intergenic region between the original G and L genes. The resulting plasmid was designated pVSV*ΔG(NP), in which the asterisk denotes the presence of the eGFP gene and ΔG indicates the absence of the glycoprotein G gene. For the CSFV vector, the NP gene was inserted into the NotI site of pA187-N^pro^-IRES-C-delE^rns^ replicon plasmid immediately downstream of and in frame with the N^pro^ autoprotease gene, as described earlier^30^.

### Generation of VRP

VSV-based VRP were generated as described previously^27,40^. Briefly, BHK-G43 cells were infected with recombinant MVA-T7 expressing T7 RNA polymerase^30^ and subsequently transfected with a plasmid carrying a VSV anti-genomic cDNA, along with three plasmids encoding the VSV proteins N, P, and L. Expression of the VSV G protein was induced with mifepristone (Sigma-Aldrich). At 24 h post transfection, the cells were harvested and mixed with an equal number of fresh BHK-G43 cells and incubated for another 24 h in the presence of mifepristone. The cell culture supernatant was clarified by low-speed centrifugation and passed through a 0.20 μm pore-size filter and titrated on BHK-21. Infectious titers were expressed as fluorescent focus-forming units (FFU) per milliliter. Functional expression of NP was tested by infecting BHK-21 cells with VSV VRP at a multiplicity of infection (m.o.i) of 1 TCID_50_/cell for 16h followed by staining with anti-NP monoclonal antibody as described above (Fig. 1a). The CSFV-derived VRP (CSF-VRP) were produced essentially as extensively described elsewhere^30,41^. Briefly, plasmids were linearized at the SrfI site and RNA run-off transcripts were generated using the MEGAscript T7 kit (Ambion). SK-6(E^rns^) cells were then transfected by electroporation with 1µg replicon RNA and the VRP harvested after 72 h. The CSF-VRP were further propagated in SK-6(E^rns^) and the infectious titre determined by end-point dilution and expressed in 50% tissue culture infectious doses (TCID_50_) per milliliter. Functional expression of NP was tested by infecting SK-6 cells with CSFV VRP at a m.o.i of 5 TCID_50_/cell for 24h followed by staining with anti-NP monoclonal antibody as described above (Fig. 1b).

### Animal experiments

The animal experiments were performed according to local law and were approved by the Ethical Committee for Animal Experiments of the Canton of Bern, Switzerland (license #BE7/12). In total, 31 healthy seven-week-old Swiss Large White pigs (18 castrated males and 13 females) from our specific-pathogen-free (SPF) breeding facility were used. The animals were housed in groups of ≥3 inside the containment facility of the Institute of Virology and Immunology IVI representing a BSL3-Ag facility. Prior to infection, the animals were allowed to accustom to the new environment for one week.

For the vaccination/challenge experiment, animals were vaccinated with either the control vectors (total n = 8; VSV*ΔG n = 5 and CSFVΔE^rns^ = 3), VSV*ΔG-NP (n = 10), CSFVΔE^rns^-NP (n = 5) or WIV (n = 5) into the gluteal and deltoideal muscle, followed by a boost four weeks. A dose of 10^8^ TCID_50_ per animal was employed for the VSV and 10^7^ TCID_50_ for the CSFV VRP in a volume of 4 ml was employed for each injection. The inactivated vaccine was formulated with 15% Montanide 15AVG (kindly donated by Seppic, Puteaux, France) and applied intramuscularly in a total volume of 4 ml. Animals were bled weekly and sera were frozen at −20 °C as described above. After four weeks, an identical booster vaccination was performed. Animals were examined clinically at daily intervals and blood was taken once a week. Three weeks after the boost vaccination, all pigs were infected via the intratracheal route with 7 * 10^6^ TCID_50_ of the heterologous virus A/swine/Bakum/R757/2010 (H1N2) (R757/10) under general anesthesia with 0.5 mg/kg Midazolam (Roche, Reinach, Switzerland) and 10 mg/kg Ketamin (Provet, Lyssach, Switzerland). Following challenge infection, all animals were examined clinically at daily intervals. The parameters recorded included body temperature, awareness, appetite, manure excretion, breathing, coughing, skin color, and gait. Blood and oronasal swabs were taken daily. At 24 h p.i., each animal was bronchoscopied and bronchoalveolar lavage (BAL) fluid was collected from both lung lobes using 20 ml PBS for rinsing under general anaesthesia. At day three post challenge infection, the pigs were euthanized by electrical stunning and subsequent exsanguination. Sampling was performed immediately after exsanguination and included swabs, blood, as well as organs for RT-qPCR analysis and histology. Three animals were treated in the same way but were neither vaccinated nor infected. The results from this manuscript were performed in two separate animal experiments. Experiment 1, was composed of four groups, including VSV*ΔG empty vector (n = 5), VSV*ΔG-NP (n = 5), WIV (n = 5) and no vaccination/no infection (n = 3). Experiment 2 had three groups, VSV*ΔG-NP (n = 5), CSFVΔE^rns^ empty vector (n = 3) and CSFVΔE^rns^-NP (n = 5).

### Virological analyses

From each animal, tissues from all 4 cranial lobes and a bronchial lymph node were collected into 1.5 ml tubes containing 500 μl MEM medium (Life Technologies), and weighed before homogenising with a BulletBlender (Next Advanced Inc. Averill Park, NY, USA). The homogenates were centrifuged and the supernatants transferred into new tubes and stored at −70 °C. RNA was extracted using the QIAmp viral RNA extraction kit (Qiagen AG, Hombrechtikon, Switzerland) according to the manufacturer’s instructions. The IAV genomic RNA segment 7 was amplified by reverse transcription and quantitative polymerase chain reaction (RT-qPCR) using primers and probe as described^42,43^. The RT-qPCR was performed as published using the SuperScript III Platinum One-Step RT-qPCR Kit (Life Technologies) and run on a 7900HT Thermocycler (Applied Biosystems) for 50 cycles. Mean Cq values were determined from triplicates. For absolute RNA quantification, an internal standard was used based on M1 genome copies^42,43^.

### Serological assays

The amounts of anti-NP antibodies in sera were determined with the FLUACA anti NP-ELISA (ID.VET, Grabels, France) according to the manufacturer’s instructions. Sera were diluted by serial two-fold dilution steps starting with a 1/25 dilution. An OD ratio (serum to negative control) of above 50% was interpreted as negative while a ratio below 45% was considered as positive. Titers were determined as the highest dilution resulting in a positive reaction. For detection of antibodies that specifically bind to NP expressed by infected cells, MDCK cells were infected for 12 h with Belzig (H1N1) or with R757 (H2N1) at an m.o.i. of 1 TCID_50_/ cell, fixed, permeabilized and incubated with the immune sera diluted at 1:200, and subsequently with fluorochrome-conjugated goat anti-pig IgG antibody (Bethyl, Montgommery, Tx, USA). The cells were analyzed by flow cytometry (FACSCalibur, BD Biosciences, Allschwil, Switzerland).

### Histopathology

For histological examination, cranial lobes were fixed in 10% buffered formalin, processed routinely for paraffin embedding, cut at 5 µm, and stained with haematoxylin and eosin (H&E). A board-certified pathologist scored all histological sections in a blinded manner. Bronchi and bronchioles were scored for epithelial necrosis, fibrin exudation and infiltration with neutrophils. Alveoli were scored for epithelial necrosis, fibrin exudation, hemorrhages, neutrophilic infiltration, thickening of the alveolar walls, and atelectasis. The following scores were given to each of the above-mentioned findings: 0 = no lesions; 1 = very mild lesions (less than 5 % of structures affected), 2 = mild lesions (5–20% of structured affected), 3 = moderate lesions (20–40% of structures affected); 4 = severe lesions (40–60% of structured affected); 5 = very severe lesions (more than 60% of structures affected). All scores were summed up to a total microscopic lung score.

### T cell assays

Frozen PBMC were thawed and cultured in AIM medium Albumax (Gibco) with 2% FBS and stimulated for 15 h at 39 °C with either IAV (Belzig/01 or R757/10) using an MOI of 0.1 TCID_50_/cell or a corresponding volume of chicken allantoic fluid (CAF). Brefeldin A (eBioscience, San Diego, CA; USA) was added and incubated with PBMC for 4 more hours. Cells were first stained with the Fixable Aqua Dead Cell Stain kit (ThermoFisher, Waltham, MA, USA) before cell surface staining was performed with anti-CD4 IgG2b (74-12-4) and anti-CD8β IgG2a (PG164A; VMRD, Pullmann, WA, USA) primary antibodies and isotype-specific AlexaFluor-488 and PE-Cy7 fluorochrome conjugates (ThermoFisher and Abcam, respectively) as secondary reagents. After fixation and permeabilization, anti-IFNγ-PE (P2G10, BD Biosciences) and anti-TNF-AF647 (Mab11, Biolegend, San Diego, CA, USA) antibody conjugates were added and cells analysed by flow cytometry (FACSCanto). Dead cells were excluded, followed by doublet discrimination, and gating on CD4 and CD8 and single positive cells to determine their intracellular IFNγ and TNFα expression.

### Statistical analysis

Analysis was performed in Prism 7 (GraphPad, USA). Data from samples obtained from both animal experiments were analyzed together as all both experiments were performed under identical conditions. The piglets were from the same breeding unit and identical age. The same vaccine and challenge virus stocks were employed. We also pooled the data from both empty vector controls as we found no evidence for an vector effect. Normality of the datasets was verified using the Shapiro-Wilk test. Statistical significance was calculated using the unpaired one-way ANOVA test, followed by Tukey’s multiple comparisons test. P-values were defined as follows: ***P < 0.0001; **P < 0.005; *P < 0.05.

## Electronic supplementary material

Supplementary Figure 1
