# Supplementary material for: Virus replicon particle vaccines expressing nucleoprotein of influenza A virus mediate enhanced inflammatory responses in pigs
Source: Sci Rep. 2017 Nov 27;7:16379. doi: 10.1038/s41598-017-16419-w (PMC5703990; doi:10.1038/s41598-017-16419-w)
Supplement: Supplementary file 1 — Supplementary Figure 1 [file 41598_2017_16419_MOESM1_ESM.doc]

**Virus replicon particle vaccines expressing nucleoprotein of influenza A virus mediate enhanced inflammatory responses in pigs**

Meret E. Ricklin^1^, Sylvie Python^1^, Nathalie J. Vielle^1^, Daniel Brechbühl^1^, Beatrice Zumkehr^1^, Horst Posthaus^2,3^, Gert Zimmer^1^, Nicolas Ruggli^1^ and Artur Summerfield*^1, 3^

^1^Institute of Virology and Immunology IVI, Sensemattstrasse 293, Mittelhäusern, Switzerland

^2^Institute for Animal Pathology, Vetsuisse Faculty, University of Bern, Länggasstrasse 122, Bern, Switzerland

^3^Department of Infectious Diseases and Pathobiology, Vetsuisse Faculty, University of Bern, Länggasstrasse 122, Bern, Switzerland

*** Corresponding author:**
artur.summerfield@ivi.admin.ch

#
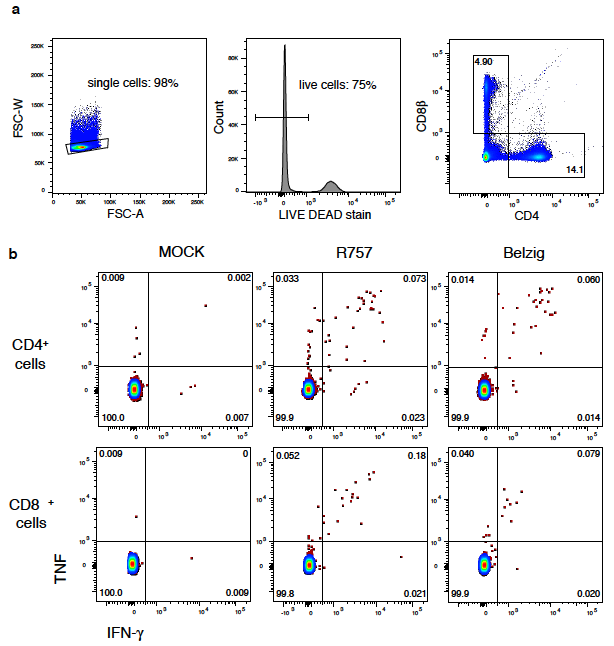


**Supplementary Figure 1.** **Representative plots showing the INFγ and TNF responses of CD4 and CD8 T-cell responses induced by NP vaccination.** PBMC from a pig vaccinated with CSFVΔE^rns^-NP were re-stimulated influenza virus as described in Materials and Method and tested for expression of CD4, CD8, IFNγ and TNF. In (**a**), the gating strategy is shown which included doublet exclusion, dead cell exclusion and CD4/CD8β gating. In(**b**) representative dot plots for IFNγ/TNF staining are shown for CD4^+^ (upper row) and CD8^+^ (lower row) cells. The cells were either restimulated with MOCK (CAV), R757 (H1N1) or Belzig (H1N1) virus.
